# Severe Hypertension in Pregnancy: Progress Made and Future Directions for Patient Safety, Quality Improvement, and Implementation of a Patient Safety Bundle

**DOI:** 10.3390/jcm13174973

**Published:** 2024-08-23

**Authors:** Alissa Prior, Isabel Taylor, Kelly S. Gibson, Christie Allen

**Affiliations:** 1 Division of Maternal Fetal Medicine, Department of Obstetrics and Gynecology, The MetroHealth System, Cleveland, OH 44109, USA; 2 Division of Maternal Fetal Medicine, Department of Obstetrics and Gynecology, University Hospitals, Cleveland, OH 44106, USA; 3 American College of Obstetricians and Gynecologists, Washington, DC 20024, USA; callen@acog.org

**Keywords:** hypertension, pregnancy, alliance for innovation on maternal health, quality improvement

## Abstract

Hypertensive disorders of pregnancy account for approximately 5% of pregnancy-related deaths in the United States and are one of the leading causes of maternal morbidity. Focus on improving patient outcomes in the setting of hypertensive disorders of pregnancy has increased in recent years, and quality improvement initiatives have been implemented across the United States. This paper discusses patient safety and quality initiatives for hypertensive disorders of pregnancy, with an emphasis on progress made and a patient safety tool: the Alliance for Innovation on Maternal Health’s Severe Hypertension in Pregnancy patient safety bundle. Future patient safety and quality directions for the treatment of hypertensive disorders of pregnancy will be reviewed.

## 1. Introduction

The prevalence of hypertensive disorders of pregnancy (HDPs), which encompass both chronic and pregnancy-associated hypertension, increased in the United States between 2017 and 2019 [1]. Furthermore, HDPs accounted for 4.9% of pregnancy-related deaths in the United States in 2020 [2], and a report from nine Maternal Mortality Review Committees found that most pregnancy-related deaths due to preeclampsia and eclampsia occurred between birth and 42 days postpartum [3]. Racial and ethnic disparities persist in rates of HDPs and associated outcomes. Black, American Indian, and Alaska Native women have a higher prevalence of HDPs compared to White women [1], and maternal mortality rates due to HDPs are higher among Black women than White women [4].

Quality improvement initiatives, or structured, collaborative approaches to improving healthcare quality, can address the quality of care for patients with HDPs and reduce associated morbidity and mortality. For example, the Ohio Perinatal Quality Collaborative improved timely treatment of persistent severe hypertension and receipt of timely follow-up appointments through collaborative-based quality improvement techniques [5]. These improvements were seen for both non-Hispanic White and non-Hispanic Black pregnant and postpartum people [5]. The Illinois Perinatal Quality Collaborative implemented quality improvement strategies to improve care for patients with HDPs, ultimately achieving reductions in severe maternal morbidity [6]. Other quality improvement initiatives achieved improvements in postpartum hypertension visit attendance and blood pressure control postpartum [7], as well as reduced racial disparities in attendance at follow-up visits for HDPs [8].

Undergirding these improvements in care and outcomes for people with HDPs is the availability of evidence-informed, updated quality improvement tools. The Alliance for Innovation on Maternal Health (AIM) developed a Severe Hypertension in Pregnancy (SHTN) patient safety bundle (PSB) in 2016 to improve the quality of care and reduce morbidity and mortality associated with HDPs. This PSB serves as a collection of evidence-informed best practices that can be implemented across care settings to improve the quality of care and health outcomes for people with hypertensive disorders of pregnancy. As of August 2023, the AIM SHTN PSB has been implemented by 34 state and jurisdiction quality improvement teams. Along the way, there have been lessons learned and opportunities identified to improve the patient safety bundle to align with emerging best practices. Additionally, the need to integrate respectful, equitable, and supportive care into all care processes, and not as a separate endeavor, has been recognized.

In response to emerging data and the focus on health equity, the original PSB was revised in 2022. The updated bundle reflects lessons learned and updates in clinical practices and incorporates respectful, equitable, and supportive care. We plan to describe the innovations in research in relation to HDPs and the purpose of the revisions to the AIM SHTN PSB to address continued quality improvement needs. In addition, we will report future directions in patient safety and quality improvement for HDPs.

## 2. Background

HDPs are a robust area of research in obstetrics, and there have been many advances in monitoring, prevention, and treatment of disease. The emerging literature on the implementation of bundles is also critical to successfully reducing morbidity [9]. Implementation science aims to bridge the divide between the development of evidence-based interventions and real-world utilization [10]. It is the scientific study of methods to promote the systematic uptake of research findings and other evidence-based practices into routine practice, and the primary goal is to improve the quality and effectiveness of health services [10]. Further, implementation science supports clinicians and policy makers in determining methods to supply tools that show success into the hands of the end users to accelerate the impact of important research findings [9]. While there are more than 60 frameworks and more than 50 strategies for implementation science, the most common framework is known as the Consolidated Framework for Implementation Research (CFIR) and has been used in studies evaluating the implementation of obstetric bundles for hemorrhage and blood pressure monitoring [9].

Standardized practices and policies, as part of implementation science, have been shown to improve safety and quality throughout many industries, including healthcare [11]. Tools have been developed to improve patient safety and quality of care, such as PSBs [7,12,13]. Pioneered in the early 2000s as a method to address Intensive Care Unit patient complications such as ventilator-associated pneumonia and central-line bloodstream infections, PSBs have shown significant success in improving patient outcomes [14]. PSBs were described by initial innovators as “…a small set of evidence-based interventions for a defined patient population and care settings” [15]. It should be noted that the initial concept of PSBs rarely includes new evidence or practices but most often identifies practices many clinicians assume are already taking place in settings of care. By implementing these sets of best practices, data may be collected to identify gaps in best-practice care and provide readily accessible ways to improve these gaps [15].

As recognition of maternal mortality and severe maternal morbidity (SMM) rose in the United States, a federal commitment was made by the Health Resource and Services Administration in 2014 to support the application of the PSB concept to obstetric care. This commitment resulted in funds awarded to the American College of Obstetricians and Gynecologists (ACOG), and the Alliance for Innovation on Maternal Health (AIM) was founded. The primary goal of AIM is to support the development and implementation of PSBs to address specific clinical conditions that were identified as leading causes of maternal mortality and SMM in the United States [16]. These PSBs could then be implemented by healthcare facilities, often with support from state or jurisdiction quality improvement teams, to improve processes of care and outcomes.

The second developed AIM PSB was Severe Hypertension in Pregnancy. After a literature review, AIM staff at ACOG convened multidisciplinary experts in obstetric care to identify best practice PSB “elements” following a “4 R” framework of Readiness, Recognition, Response, and Reporting and Systems Learning (Table 1). Following the identification of key elements, a Data Collection Plan (DCP) was designed to allow implementing quality improvement teams to benchmark and measure progress. Both the PSB and DCP were made publicly available in 2015. A Consensus Statement on Severe Hypertension in Pregnancy PSB was published in 2017 [17].

## 3. Revision of the Severe Hypertension in Pregnancy Patient Safety Bundle to Address Developments in the Field

In response to current evidence and best practices, as well as lessons learned, the AIM program revised its SHTN PSB in 2022. Interdisciplinary experts convened to review the existing PSB and draft revisions based on current clinical practice and evidence, use of inclusive language, and integration of respectful care concepts. The revised PSB was then reviewed by external experts and organizations, including patients with lived expertise, for feedback and refinements before finalization of the revised PSB.

Broadly, revisions to the SHTN PSB focused on activities to improve the recognition of and response to HDPs, particularly in the prenatal and postpartum time periods and in settings outside of labor and delivery, timely treatment of persistent severe hypertension, and improvements in care transitions in the early postpartum period. Additionally, actionable strategies for the integration of respectful, equitable, and supportive care into practice, which were informed by respectful maternity care frameworks and other data, were included in the revised PSB.

### 3.1. Theme 1: Respectful, Equitable, and Supportive Care

Racial and ethnic disparities in maternal health outcomes are marked. In 2022, maternal mortality rates for non-Hispanic Black people were around 2.6 times higher than non-Hispanic White people [18], and in 2020, maternal mortality rates were 3.5 times higher for American Indian and Alaska Native people than non-Hispanic White people [2]. SMM, or unexpected outcomes during the delivery hospitalization that result in significant short- or long-term consequences to a person’s health, are also higher among non-Hispanic Black people compared to non-Hispanic White people [19,20]. Racial and ethnic disparities in the prevalence of HDPs and associated maternal mortality rates due to HDPs have also been noted [1,4].

Integration of respectful, equitable, and supportive care into quality improvement and care processes is one way to address the inequities in care [21]. Respectful care is an approach to clinical care grounded in reproductive justice, which is a Black woman-developed concept that centers bodily autonomy autonomy and the respect and safety of individuals and communities [22]. Respectful care is multifaceted, and central components include access to care, equity, consent, and autonomy [21]. Respectful care may be supported through teamwork, communication, and informed decision making [21]. Like quality improvement, the cycle to respectful care is continuous, and efforts should be taken to continuously ground all quality improvement efforts in health equity and respectful care to address how inequities may undermine even the most robust standardization of care processes [23,24].

In 2015, a PSB was developed titled “Reduction of Peripartum Racial and Ethnic Disparities,” which identified actionable steps that could be taken using a quality improvement approach to reduce inequities in care and disparities in outcomes [25]. While separate from the other AIM PSBs focusing on clinical conditions contributing to SMM and maternal mortality, innovations were made in “overlaying” clinical condition-specific AIM PSBs with the Reduction of Peripartum Racial and Ethnic Disparities PSB to improve quality of care in a manner grounded in equity and respectful care [26]. To better integrate concepts of equity into quality improvement in perinatal care, the decision was made to integrate a “5th R” of respectful, equitable, and supportive care into all AIM patient safety bundles as they were revised. These respectful care concepts were interwoven into all elements of the AIM SHTN PSB.

The core of the actionable respectful care approach includes three discrete types of interventions: screening and referral for unmet social needs via social determinant of health assessment, provider education and application of antiracist and trauma-informed care concepts, and inclusion of the patient and their support network as valued and contributing members of the care team. As with all quality improvement interventions, these concepts require monitoring of data at both baseline and in an ongoing manner to assess efficacy and achievement of desired outcomes. Data collection and monitoring elements were revised to include, at minimum, the disaggregation of race and ethnicity in implementation data. Following the drafting of the revised bundle, it was reviewed by the Preeclampsia Foundation’s Patient Advisory Council, and feedback from those with lived experience of HDPs was integrated into the revised SHTN PSB elements.

### 3.2. Theme 2: Recognition of Hypertensive Emergencies across Care Settings

People with HDPs are at increased risk of postpartum hospital encounters, including Emergency Department visits and hospital readmissions, compared to those without HDPs [27]. Among 60-day readmissions, people with HDPs are most frequently readmitted for care within 10 days after discharge from the delivery hospitalization [28]. It is important to note that hypertensive emergencies can occur at any time in the prenatal and postpartum periods, in addition to the birth hospitalization. Patients experiencing these emergencies may present for care at entry points that do not primarily provide care for obstetric patients, such as outpatient urgent care, Emergency Departments, or primary care settings. Due to the nonspecific nature of the presentation of symptoms of HDPs and unique parameters that constitute severe range hypertension in pregnancy, as well as the age and overall health status of these patients, typical screening and triage methodologies may not identify this health emergency in the perinatal population. This underscores the need for screening for current or recent pregnancies in all care settings.

Screening for current or recent pregnancy to contextualize presenting signs and symptoms remains low in Emergency Departments in the United States [29]. Further, pregnant and postpartum patients with systolic blood pressure over 140 mmHg were under-triaged in a study of five Emergency Departments [29], and presentation to the Emergency Department was associated with a lack of timely initiation of treatment of persistent SHTN in pregnant and postpartum people [30]. These considerations highlight opportunities to improve the quality of care for patients with HDPs through coordination of obstetric and Emergency Department providers to share knowledge and implement appropriate processes to recognize and escalate care for patients presenting with obstetric emergencies to non-obstetric settings.

In response, AIM has included the following bundle element in many of its revised and newly developed PSBs, including SHTN: “Assess and document if a patient presenting is pregnant or has been pregnant within the past year in all care settings” [31]. While different laws and restrictions regarding reproductive health in the United States may lead to challenges around this recommendation, screening is intended to contextualize presenting patient symptoms for appropriate escalations in care. The screening can be verbal, and care should be taken to protect patient privacy and confidentiality during screening and in documentation. It should be noted that the pregnancy or postpartum status, not the outcome or resolution of a pregnancy, is the key piece of information to be obtained from this screening [32].

Both ACOG and the Centers for Disease Control and Prevention (CDC) have resources to support Emergency Department provider recognition of pregnant and postpartum patients. ACOG co-developed with the CDC materials for non-obstetric settings, encouraging patients to disclose current or recent pregnancy status to help contextualize their care [33]. Additionally, the CDC HEAR HER^®^ Campaign has healthcare professional-facing materials to encourage screening for current or recent pregnancy [34]. These materials, combined with patient education materials on urgent signs and symptoms during pregnancy and postpartum and when to seek care from ACOG, the CDC, and other sources, can help empower patients and providers alike to recognize and respond to obstetric emergencies [35,36]. These materials have the potential to help operationalize pregnancy screening in other non-obstetric care settings.

### 3.3. Theme 3: Timely Treatment of Persistent Severe Hypertension

Evidence suggests that timely treatment of persistent severe hypertension (PSHTN), which is defined as a systolic or diastolic blood pressure of 160/110 mmHg or greater that is persistent for 15 min or longer, is associated with lower rates of SMM compared to those with PSHTN who do not receive timely antihypertensive treatment [37]. Timely treatment of PSHTN is complex, with many factors influencing implementation and measurement. Factors such as delayed blood pressure re-checks [30], patient presentation with PSHTN to the Emergency Department [30], and transitions across the continuum of care that result in a lack of recognition and escalations in care may contribute to a lack of timely treatment of PSHTN [37]. Other documented factors precluding the timely treatment of PSHTN include non-severe range hypertension on admission for care and PSHTN that occurred within 1 h of delivery [38].

Facilitators of the timely treatment of PSHTN exist, such as clinical education on the significance of PSHTN, appropriate measurement and confirmation of elevated blood pressure readings, and clinical algorithms and protocols to respond to instances of PSHTN [39]. In one study, this extensive education, combined with systems changes focused on the implementation of algorithms, protocols, and order sets and improved multidisciplinary communication resulted in significant improvements in the time to treatment for PSHTN [39].

Understanding the barriers to and facilitators of the timely treatment of PSHTN is critical, as it demonstrates sustained quality improvement needs and opportunities to appropriately care for those experiencing PSHTN. To minimize adverse outcomes, ACOG recommends treating severe hypertension (systolic blood pressure ≥160 mmHg and/or diastolic blood pressure ≥110 mmHg) that is persistent (≥15 min) as soon as reasonably possible, with a target of time to treatment within 30–60 min of recognition of PSHTN [40]. The AIM program has placed an emphasis on the timely treatment of PSHTN, supporting quality improvement implementation and adapting measurement strategies to align with lessons learned.

Initially, AIM conceptualized the timely treatment of PSHTN for quality improvement measurement as treatment with appropriate antihypertensive medication within 60 min of the second severe-range blood pressure (SRBP) reading confirming PSHTN. However, delays in follow-up blood pressure measurement may result in a further delayed treatment than if a timely treatment was measured based on the first SRBP reading [30,41].

AIM then worked with the Society for Maternal-Fetal Medicine (SMFM) Patient Safety and Quality Committee to standardize the measurement of PSHTN, choosing to measure the timely treatment of PSHTN using the first SRBP reading, thus encouraging providers to perform timely rechecks of blood pressure for confirmation of PSHTN [41]. This standardization of measurement is crucial, as it helps to prioritize timely recognition of PSHTN and escalations in care, and it supports meaningful comparisons in quality improvement collaboratives. This will help teams to address barriers to and facilitators of the timely treatment of PSHTN through a data-driven implementation science lens.

### 3.4. Theme 4: Transitions of Care

Data from Maternal Mortality Review Committees in 38 states in 2020 show that nearly half (47%) of pregnancy-related deaths occurred between 7 and 365 days postpartum [42], highlighting the importance of optimizing transitions of care or transitions from one care setting to another. ACOG recommends that patients with severe hypertension receive a blood pressure evaluation within 72 h of discharge from delivery hospitalization, and patients with other HDPs receive a blood pressure evaluation within 7 to 10 days after delivery hospitalization discharge [43]. Assessing patients with SHTN and HDPs early and frequently in the outpatient setting has several benefits, such as preventing progression to severe disease and, if inpatient evaluation is needed, more timely intervention and potential prevention of SMM and mortality [44].

Transitions of care after pregnancy for people with HDPs have been described as inadequate despite recommendations from professional societies on the value of coordination and continuity in care [45]. Studies have shown that attendance at postpartum blood pressure evaluations among those with HDPs is low [46,47] and that the quality of information shared during these meetings, particularly regarding future HDP and cardiovascular disease risk, is also lacking [45].

In response to ongoing quality improvement opportunities to optimize transitions of care and the associated potential to improve health outcomes, the AIM SHTN PSB was revised to state, “Initiate postpartum follow-up visit to occur within 3 days of birth hospitalization discharge date for individuals whose pregnancy was complicated by HDPs” [31]. The parameter of three days was selected by the expert revision work group due to data on the timing of pregnancy-related deaths, as HDPs are among the leading causes of pregnancy-related death up to 42 days postpartum [3]. This timing was of particular interest to the working group in the setting of the COVID-19 pandemic, which occurred during the bundle revision and caused disruption to typical admission length and follow-up care for postpartum people.

The goals of this postpartum in-person or telehealth follow-up visit include a blood pressure check, a discussion of signs and symptoms of hypertensive emergencies, who to contact regarding worsening symptoms of hypertension, and where to go in case of an emergency [48]. Due to documented low rates of attendance at these visits, proactive patient education on the value of early postpartum blood pressure and symptom checks, as well as identification of barriers and unmet social needs that may make attendance at these visits more difficult, should be implemented and addressed.

Quality improvement initiatives focused on improving postpartum transitions in care for people with HDPs have shown improvements in the coordination of and attendance at early postpartum visits for people with HDPs [5,7], as well as reductions in racial disparities in attendance at these visits via the implementation of telehealth [8]. Ongoing prioritization of these early postpartum visits and the development of coordinated care pathways from AIM, as well as future work in remote blood pressure monitoring programs and the role of telehealth, may further enhance postpartum care transitions for people with HDPs on a national scale. 

## 4. Future Directions

**Figure 1:**
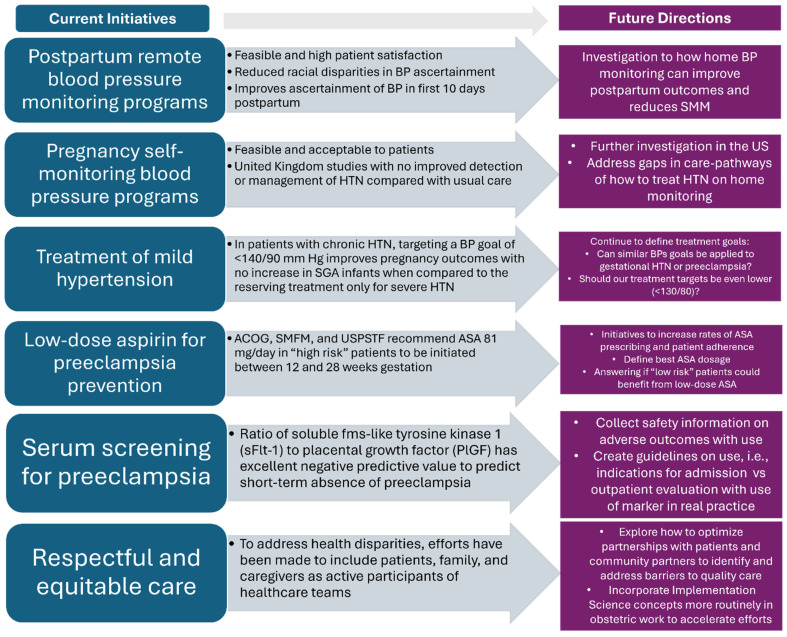
Summary of current initiatives and future directions for care of people with hypertensive disorders of pregnancy.

There are several future directions for the monitoring, screening, and treatment of HDPs (Figure 1). Research has been dedicated to postpartum remote blood pressure monitoring programs, with promising results. A prospective observational cohort study of postpartum patients with HDP enrolled in a remote, text message-based blood pressure monitoring program showed this is a feasible intervention with reported high patient satisfaction [49]. In a study across three US academic medical centers, 97% of patients submitted at least one blood pressure measurement via text message, with 86% submitting blood pressures on postpartum days 7 to 10 and 67% on days 1 to 4. Further, a systematic review of thirteen studies that evaluated the effects of postpartum home blood pressure monitoring on maternal and infant outcomes revealed this intervention improves ascertainment of blood pressure in the first 10 days postpartum [50]. Home blood pressure monitoring, compared with office-based follow-up, was also associated with reduced racial disparities in blood pressure ascertainment by ~50% [50]. There was insufficient evidence to conclude that home blood pressure monitoring reduces SMM.

Self-monitoring blood pressure (SMBP) programs during pregnancy have also been investigated in the last five years. Early small observational studies showed that SMBP programs are acceptable and feasible for patients [51,52]. In the United Kingdom’s Blood Pressure Monitoring in Pregnancy (BUMP) trials, two randomized controlled trials of SMBP in pregnancy involving over 3000 patients at higher risk of preeclampsia were randomly allocated to either usual care or usual care plus SMBP [53]. Although these studies showed SMBP during pregnancy is safe, feasible, acceptable to patients, and no more expensive than usual care, it did not improve the detection or management of hypertension compared with usual care [53]. Home blood pressure programs should be further investigated in the United States, and gaps in care pathways regarding how to treat hypertension through home monitoring need to be addressed.

Thresholds for the management of blood pressure in pregnancy also present an area for future directions in care. Major insights into the treatment of mild hypertension in pregnancy arose from the Chronic Hypertension and Pregnancy (CHAP) project. This multicenter, randomized controlled trial performed at 70 sites in the United States was undertaken to investigate the benefits and safety of treatment of mild chronic hypertension during pregnancy [54]. Due to concerns that antihypertensive treatment in pregnancy was associated with increased risk for small-for-gestational-age birth weight [55,56,57], recommendations for treatment of mild chronic hypertension (typically defined as blood pressure <160/110 mmHg) were unclear. In this trial, women with singleton fetuses and mild chronic hypertension at <23 weeks gestation were randomized into the active-treatment group to receive antihypertensive medications to a goal blood pressure of <140/90 mmHg versus the control group, where treatment was only initiated for severe hypertension. The primary outcome, a composite of preeclampsia with severe features, medically indicated preterm birth at less than 35 weeks’ gestation, placental abruption, or fetal or neonatal death, was significantly lower in the active-treatment group. The primary safety outcome of poor fetal growth, defined as a birth weight less than the 10th percentile, was not significantly different between treatment and control groups. It was concluded that in pregnant patients with mild chronic hypertension, targeting a blood pressure of less than 140/60 mmHg was associated with better pregnancy outcomes, with no increase in the risk for small-for-gestational-age birth weight, when compared with the previous strategy of reserving treatment only for severe hypertension.

Since this publication, professional societies such as ACOG and SMFM have released statements supporting the initiation of antihypertensive therapy to maintain blood pressure below 140/90 mmHg for pregnant patients with chronic hypertension [56,58]. While some studies have suggested further benefits with a lower treatment goal, such as 130/80, or the treatment of patients with AHA Stage I hypertension, the only available data are retrospective [59,60]. More research is needed to determine whether the blood pressure goal should be lowered; how these guidelines should be used with other hypertensive diseases of pregnancy, including gestational hypertension and preeclampsia; and if treatment initiation is safe and warranted in the third trimester.

Prevention of preeclampsia with low-dose aspirin during pregnancy has become a cornerstone of practice in prenatal care. ACOG, SMFM, and the United States Preventive Services Task Force (USPSTF) currently recommend that 81 mg/day of aspirin in people at high risk of preeclampsia be initiated between 12 weeks and 28 weeks of gestation and continued until delivery [61,62,63]. Despite clear recommendations and established risk criteria for aspirin prophylaxis, challenges to the implementation of this guideline include providers performing a baseline risk assessment during routine prenatal care, appropriate prescribing, communication between patients and providers about the recommendations, and patient adherence to prophylaxis. Quality improvement initiatives have been successful in improving uptake, such as the implementation of preeclampsia risk screens in the electronic health record to ensure standardized screening, leading to improved prescribing practices [64].

Other research efforts have investigated the effect of aspirin dose on the incidence of preeclampsia. Major societies outside of the United States, including the Royal College of Obstetricians and Gynecologists and the National Institute of Health and Care Excellence, recommend 150 mg of aspirin daily, specifically in those at high risk of preeclampsia. One meta-analysis reported that higher aspirin doses were associated with reductions in preterm preeclampsia, although the findings were limited by the heterogeneity of the data [65]. Randomized controlled trials examining 81 mg vs. 162 mg of aspirin daily for the prevention of preeclampsia are needed in the U.S. to assess the safety and efficacy of currently available aspirin dosages.

In addition to preeclampsia prevention, there is a focus on the development of serum screening for the disease. The measurement of proteinuria can be prone to inaccuracies; therefore, serum markers to support the diagnosis of preeclampsia have been investigated. In 2016, a prospective, multicenter, observational study analyzed the use of the ratio of soluble fms-like tyrosine kinase 1 (sFlt-1) to placental growth factor (PlGF) [64]. This study showed an sFlt-1:P1GF ratio of 38 or lower has an excellent negative predictive value to predict the short-term (7-day) absence of preeclampsia in women in whom the syndrome is suspected clinically [66]. Further investigation of this serum marker, safety information on adverse outcomes with use in pregnancies, and guidelines on parameters for patient admission or outpatient evaluation of suspected preeclampsia are needed.

An emphasis on respectful and equitable care, along with addressing health disparities, is a principal area of QI work. One component of equitable care is the involvement of patients, family, and caregivers as active participants in the healthcare team [10,67,68]. AIM has worked to integrate patients and families with lived experience into PSB implementation and quality initiatives. Patients provide valuable insights into areas for improvement, including reducing unnecessary interventions, improving access to services, and improvement in communication strategies. Future research in this area is needed to explore how to optimize partnerships with patients and community partners to identify and address barriers to quality care and reduce SMM in obstetric patients.

Finally, IS continues to be another promising avenue to turn evidence into practice [10]. The field is organized through models, theories, and frameworks to aid in identifying why an intervention succeeds or fails. Recently, the field has also focused on addressing health disparities and the benefit of community partnerships with implementation [69]. It is often referenced that, on average, the gap from publication to regular incorporation into practice is 17 years [70,71]. This field considers the patient, provider, organization, and health policy which impacts intervention implementation [72]. Incorporating IS concepts more routinely into obstetric work may accelerate future directions and efforts to reduce maternal mortality and SMM in HDP.

## 5. Conclusions

Overall, HDPs remain one of the leading causes of maternal morbidity in the United States despite an abundance of research and quality efforts to improve care. This highlights continued opportunities to turn evidence into routine, standard practice. Existing quality improvement tools, such as AIM patient safety bundles, seek to turn evidence into practice and provide a standardized approach to care. The AIM SHTN PSB was revised to reflect updates in research and lessons learned from ongoing quality efforts to improve care for people with HDPs, with a focus on improving recognition and response to hypertensive emergencies through the incorporation of respectful, equitable, and supportive care; screening for current and recent pregnancy in all care settings; timely treatment of PSHTN; and improved postpartum transitions of care. Future patient safety tools that include considerations focused on the prevention of HDP in addition to the timely recognition of and response to severe hypertension is a promising future direction in care.

## Figures and Tables

**Table 1:** Example elements from the revised Severe Hypertension in Pregnancy patient safety bundle.

Patient Safety Bundle Domain	Example Patient Safety Bundle Elements
Readiness	Ensure rapid access to medications used for severe hypertension/eclampsia with a brief guide for administration and dosage in all areas where patients may be treated.
Recognition and Prevention	Provide ongoing education to all patients on the signs and symptoms of hypertension and preeclampsia and empower them to seek care.
Response	Utilize a standardized protocol with checklists and escalation policies, including a standard response to maternal early warning signs, listening and investigating patient-reported and observed symptoms, and assessment of standard labs for the management of patients with severe hypertension or related symptoms.
Reporting and Systems Learning	Monitor outcomes and process data related to severe hypertension, disaggregated by race and ethnicity due to known disparities in rates of severe hypertension.
Respectful, Equitable, and Supportive Care	Engage in open, transparent, and empathetic communication with pregnant and postpartum people and their identified support network to understand diagnoses, options, and treatment plans.
